# Education Research: Has Video Killed the Interview Star? A Survey of Current Neurology Residents on In-Person vs Virtual Residency Interviews

**DOI:** 10.1212/NE9.0000000000200161

**Published:** 2024-10-31

**Authors:** William Alexander Dalrymple, Robin Ulep, Jeffrey B. Ratliff, Joseph Carrera, Alan Wang, James T. Patrie, Andrew M. Southerland

**Affiliations:** From the Department of Neurology (W.A.D., A.M.S.), University of Virginia, Charlottesville; Department of Neurology (R.U.), Icahn School of Medicine at Mount Sinai, New York, NY; Department of Neurology (J.B.R.), Thomas Jefferson University, Philadelphia, PA; Department of Neurology (J.C.), University of Michigan Medical School, Ann Arbor; Department of Neurology (A.W.), University of Arizona College of Medicine – Phoenix; and Department of Public Health Sciences (J.T.P., A.M.S.), University of Virginia, Charlottesville.

## Abstract

**Background and Objectives:**

The residency application process relies on interviews, which allow programs and applicants to assess one another. Historically, interviews were conducted in person at each program. With the advent of the coronavirus disease 2019 pandemic, residency interviews shifted to a virtual format. Now, many specialties are choosing to return to in-person interviews. The objective of this study was to evaluate the resident perspective of virtual and in-person interviews.

**Methods:**

We created a survey about various aspects of the residency interview process and distributed it to neurology residents in all years of training from 5 institutions across the United States. Because of the timing of survey distribution, some residents interviewed in-person while others interviewed virtually. We focused the survey on a few themes: number of applications, cost, and overall quality. Survey response data were analyzed using generalized linear models and by nonparametric methods for categorical data.

**Results:**

Of the 164 total residents among the 5 programs, 60 completed the survey; 25 (41.7%) interviewed in-person while 35 (58.3%) interviewed virtually. Applicants who interviewed virtually applied to more programs (38.2 ± 26.6 vs 20.7 ± 7.4, *p* < 0.001) and attended more interviews (15.4 ± 8.3 vs 11.6 ± 3.3) but received a lower percentage of interview offers (54.3% ± 23.0% vs 74.4% ± 19.8%). Applicants who interviewed in-person spent significantly more money (95% CI $2,000–3,500 vs $15–100) but were also more confident in their assessment of a program's culture (76.9% vs 17.1%) and location (56.0% vs 8.6%). When asked which method they would prefer, respondents chose the method that they were familiar with—96% of people who interviewed in-person would prefer in-person interviews while 68.6% of those who interviewed virtually would prefer virtual interviews (*p* < 0.001).

**Discussion:**

There are multiple factors to consider when deciding on in-person or virtual residency interviews. In-person interviews are significantly more expensive and thus raise issues of equity but also provide better insight into the culture, location, and “fit” of programs and can help to reduce application burden. All these factors need to be considered before moving forward with a decision on residency interview formats for the future.

## Introduction

Interviews are a crucial part of the application and selection process for medical residencies. Historically, applicants invited for interviews would travel to the program, where they would meet with current residents before undergoing a series of interviews and other activities. With the advent of the coronavirus disease 2019 (COVID-19) pandemic in 2020, this tradition of in-person residency interviews was upended. Over the ensuing few years, both programs and applicants adapted to a new interview format, with many programs offering virtual approximations of the interview day. A previous survey of fourth-year medical students in 2020–2021 found that, although 71.7% of students felt that they could “represent themselves to the program” in a virtual format, only 46% “confidently believed they could assess their fit into the program.”^1^ Notably, the previous study also showed that most respondents found virtual interviews to have significant financial benefits compared with in-person interviews.^1^

Now, with the COVID-19 pandemic largely subsiding, programs are left with a choice of whether to continue with virtual interviews or return to in-person. The American Academy of Neurology consensus statement for the 2023–2024 interview season advised all programs to conduct virtual interviews, aligning with a similar recommendation from the American Association of Medical Colleges to “maintain an equitable interview process for all candidates.”^2^ Applicants themselves may find that they have little voice in this decision. We sought to gather opinions from current neurology residents on this very issue. As recent applicants who have collectively interviewed both in-person and virtually, they are uniquely situated to provide candid views on the pros and cons of both interview formats.

## Methods

### Survey and Participants

We at the University of Virginia (UVA) School of Medicine teamed with 4 other partner sites (Sidney Kimmel Medical College at Thomas Jefferson University, University of Michigan School of Medicine, Icahn School of Medicine at Mount Sinai, and University of Arizona College of Medicine), chosen for a mix of geographic diversity (southeast, northeast, midwest, and west; large and small cities) and convenience, as the authors have previously collaborated. A summary of the major characteristics of each neurology residency program is presented in Table 1. Together, we created an electronic survey investigating various aspects of the residency interview process. All authors contributed to development of the survey, and it underwent multiple revisions but no pilot testing or validation before finalization. The final survey consisted of 27 questions, broadly split across 4 themes: interview basics, economic and environmental burdens, second looks, and overall quality (eAppendix 1). Notably, we omitted demographic questions from the survey because it could have potentially led to a lack of anonymity among the respondents. Once the survey questions were finalized, the survey was distributed to all neurology residents at these programs by email in August 2023, with data collection finishing in October 2023.

**Table 1:** Summary of Key Characteristics of Participating Programs

Program	Location	Setting	Residents/year
University of Virginia School of Medicine	Charlottesville, VA	Academic	6
Sidney Kimmel Medical College at Thomas Jefferson University	Philadelphia, PA	Academic	9–11
Icahn School of Medicine at Mount Sinai	New York, NY	Academic	10
University of Michigan School of Medicine	Ann Arbor, MI	Academic	9–13
University of Arizona College of Medicine	Phoenix, AZ	Academic	6

### Standard Protocol Approvals, Registrations, and Participant Consents

The entire study was approved by the institutional review board (IRB) at the UVA (IRB-SBS #5737) and by the Graduate Medical Education Executive Committee at UVA for distribution to resident physicians. Data Use Agreements were completed with the partner sites, and each site also obtained their own IRB approval. Survey participation was anonymous and voluntary, and residents were given approximately 2 months to complete the survey. Informed consent was obtained from each participant before the start of the survey.

### Statistical Analysis

Survey questions that required respondents to select between categorical levels were summarized by frequencies (i.e., n) and relative frequencies (i.e., %). Survey questions with continuous scaled or count information were summarized by the mean or median of the empirical distribution and 95% confidence intervals. An α = 0.05 significance level was used as the null hypothesis rejection rule for all hypothesis tests. All statistical analyses were conducted using SAS version 9.4 (SAS Institute, Cary, NC).

A negative binomial regression analysis was conducted to compare the mean number of programs the residents applied to between residents who interviewed virtually and residents who interviewed in-person. A logistic regression analysis was conducted to compare the odds for receiving and accepting an interview offer between the 2 groups. Pearson χ^2^ exact tests were conducted to compare the response distributions of the 2 groups for questions investigating the reasons for not attending an interview when one was offered.

Interview cost was compared between the 2 groups by way of a log-rank χ^2^ test. Pearson χ^2^ exact tests were also conducted to compare the frequency of loan burden and debt between the 2 groups.

The empirical response distributions of the questions related to second looks were compared between the 2 groups by way of Pearson χ^2^ exact tests.

The empirical response distributions of the questions related to interview quality were compared between the 2 groups using Pearson χ^2^ exact tests.

### Data Availability

Full data from this study will be stored in a Research Electronic Data Capture database on the server at the UVA and will be made available on request of other researchers.

## Results

### Participants and Interview Basics

Of the 164 total residents between the 5 programs involved in the survey, 77 (47%) agreed to the informed consent document and began the survey and 60 (77.9% of those who started the survey) completed the full survey. Of the 60 residents with complete surveys, 25 (41.7%) interviewed for residency in-person and 35 (58.3%) interviewed for residency virtually.

**Figure 1:**
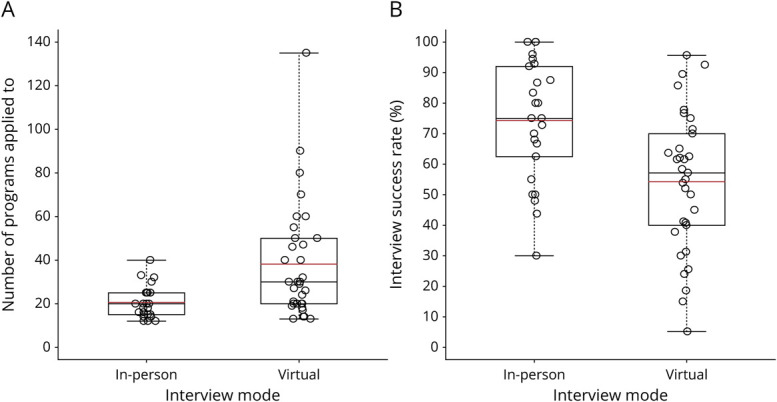
Empirical Distribution for the Number of Programs Applied to (A), and Application to Interview Offer Success Rate (B)

**Figure 2:**
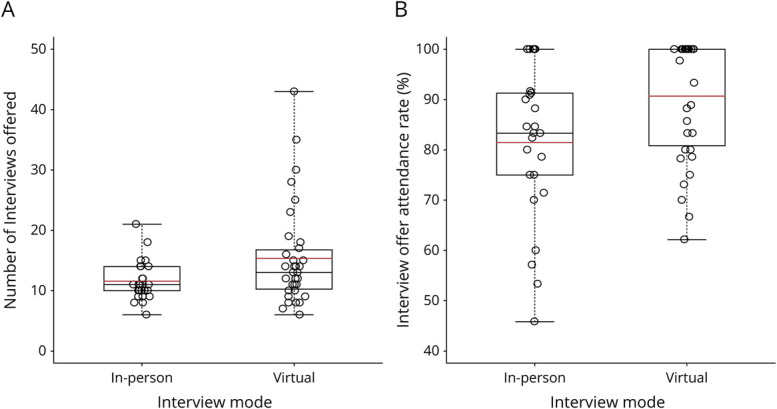
Empirical Distributions for the Number of Interviews Attended (A) and Percent of Interview Offers Attended (B)

Those who interviewed in-person applied to a mean of 20.7 programs (95% CI 16.1–25.4) while those who interviewed virtually applied to a mean of 38.1 programs (95% CI 32.1–45.4). Thus, applicants who interviewed virtually applied to nearly double the number of programs as those who interviewed in-person (ratio of means 1.84, 95% CI 1.41–2.41, *p* < 0.001). Applicants who interviewed in-person were offered a mean of 14.7 interviews, for an application to offer a success rate of 74.4%; applicants who interviewed virtually were offered a mean of 17.6 interviews, for a success rate of 54.3% (Figure 1). The odds for a resident to receive an interview offer for a given application was 2.95 times (95% CI 2.36–3.68, *p* < 0.001) greater for the residents who interviewed in-person compared with the residents who interviewed virtually. Applicants who interviewed in-person attended a mean of 11.6 interviews, or 81.5% of those they were offered, while applicants who interviewed virtually attended a mean of 15.4 interviews, or 90.7% of those they were offered (Figure 2). The odds for a resident to attend an interview was 1.85 times (95% CI 1.31–2.62, *p* = 0.001) greater for the residents who interviewed virtually compared with those who interviewed in-person.

Subsequent questions investigated the reasons for not attending an interview when one was offered. Applicants who interviewed in-person were much more likely to list cost (28% vs 2.9%, *p* = 0.004) and conflict with another interview (60% vs 11.4%, *p* < 0.001) as barriers to attending interviews (Table 2). There was no statistically significant difference between the 2 groups for other reasons for not attending interviews.

**Table 2:** Predicting Interview Mode Based on the Reason for Not Attending an Interview Offer

Predicting in-person mode of interview			
Reason for not attending	Ratio	Adjusted odd ratios (95% CI)	*p* Value
Cost	Yes:no	21.50 (1.08–428.06)	0.044
Time off rotation	Yes:no	0.42 (0.04–4.23)	0.463
Conflict with another interview	Yes:no	16.71 (2.96–94.35)	0.001
Did not like program	Yes:no	1.93 (0.44–8.43)	0.383
Significant other not offered interview	Yes:no	0.39 (0.04–4.15)	0.433
Other	Yes:no	0.36 (0.07–1.84)	0.221

### Economic and Environmental Burdens

**Figure 3:**
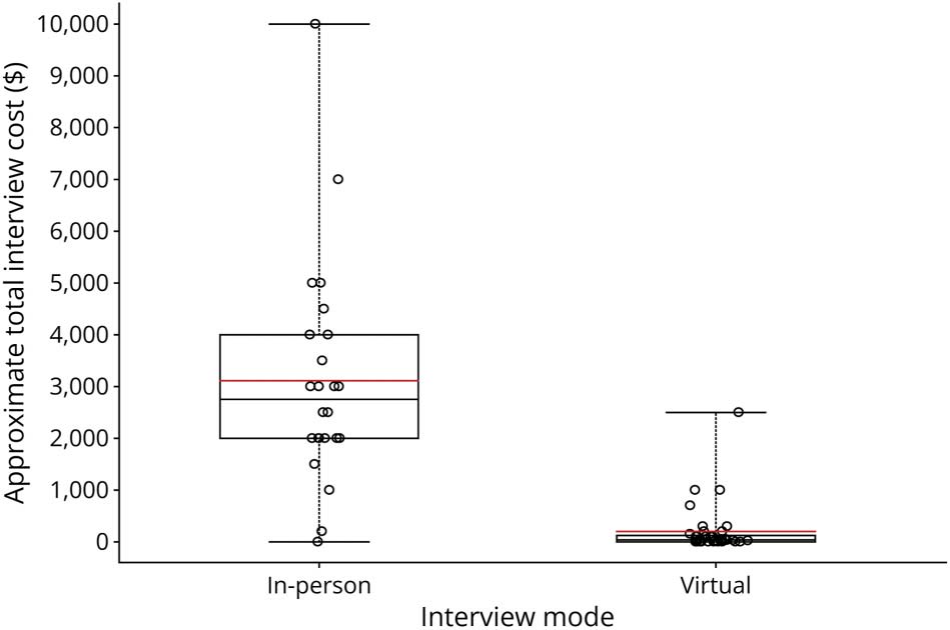
Empirical Distributions for the Approximate Total Cost of Interviews

Applicants who interviewed in-person spent a median of $2,750 on interview expenses while those who interviewed virtually spent a median of $40 on interview expenses (*p* < 0.001) (Figure 3). 28% (7/25) of the in-person applicants incurred debt to cover interview costs while only 2.9% (1/35) of the virtual applicants did the same (*p* = 0.007). Applicants who interviewed in-person drove to an average of 5.4 interviews, flew to an average of 4.3 interviews, and took public transportation to an average of 1.1 interviews. Among those who interviewed virtually, 23 (65.7%) interviewed from the same place each time.

### Residency Program Second Looks

7 of the 25 in-person applicants (28.0%) and 11 of the 35 virtual applicants (31.4%) attended second look events. Most of the second looks were in person for the in-person group (85.7%) and virtual for the virtual group (63.6%). Among those attending second looks, only 14.3% of the in-person group found the event to be very or extremely helpful in confirming their final rank list decision while 54.5% of the virtual group found them to be very or extremely helpful in confirming their final rank list decision, although this difference was not statistically significant (*p* = 0.150). There were no differences in time or cost considerations for attending second look events between the 2 groups.

### Quality

**Figure 4:**
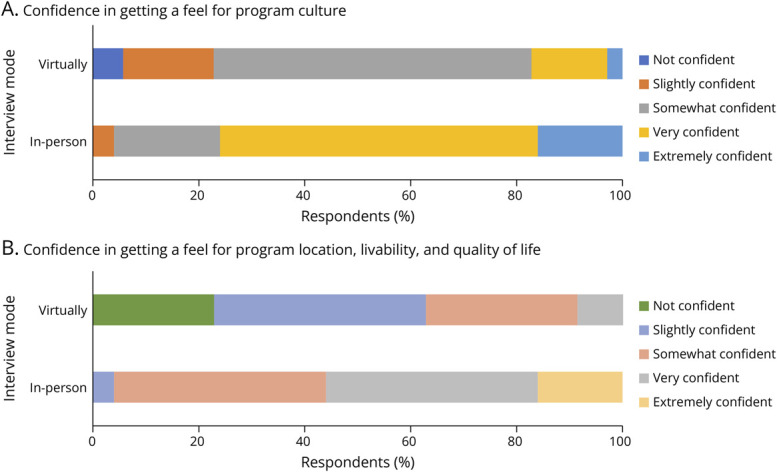
Level of Confidence With Respect to “Getting a Feel for the Culture of a Program” (A), and Level of Confidence With Respect to “Getting a Feel for the Livability and Quality of Life of the Program's Location” (B)

All respondents believed that preinterview gatherings (e.g., resident dinners or socials) were effective at informing them about the culture of a program, but the in-person group was substantially more likely to feel that these gatherings were very or extremely effective (88.0% vs 44.1%, *p* = 0.001). There were no differences between the in-person and virtual groups in their perceived ability to communicate with program leadership, other faculty, and administrative staff during the interview day. Residents who interviewed in-person were more likely to express confidence in “getting a feel for the culture of a program” (76.9% very or extremely effective vs 17.1% among virtual applicants, *p* < 0.001) and in “getting a feel for the livability and quality of life of a program's location” (56.0% very or extremely effective vs 8.6% for virtual applicants, *p* < 0.001) (Figure 4). There were no significant differences between the 2 groups regarding the overall length and content of the interview days or in effectiveness of communication before and after interviews. If given the choice, residents who interviewed in-person were overwhelmingly likely to prefer in-person interviews (96.0%) while those who interviewed virtually were more likely to prefer virtual interviews (68.6%) (*p* < 0.001 for between-group comparison). When all respondents were combined, 35 (58.3%) of 60 would choose to interview in-person (*p* = 0.245) (Table 3).

**Table 3:** If Given the Choice, Would You Prefer Interviews Be In-Person or Virtual?

	Response, n (%)	
Interview mode	In-person	Virtual
In-person	24 (96.0)	1 (4.0)
Virtually	11 (31.4)	24 (68.6)
	Fisher exact test: *p* < 0.001	
Both	35 (58.3)	25 (41.7)
	%(in-person) = %(virtual) = 50%, *p* = 0.245	

## Discussion

Among myriad other effects on medical education, the COVID-19 pandemic forced a sudden, dramatic change in the residency application process with a shift to a virtual interview format for all specialties. As the pandemic subsides, many specialties, programs, and institutions are now deciding whether to return to the in-person interviews of the past or to continue with virtual interviews. Our study investigated the pros and cons of both, by surveying those who have most recently gone through the process and have bridged the gap between the prepandemic and postpandemic environments: current neurology residents. Given the timing of the survey, we were able to obtain responses from residents who interviewed in either format.

Residents who interviewed virtually applied to far more programs than did those who interviewed in-person. The average number of interview offers, however, was only slightly different between the 2 groups, which means that residents who interviewed in-person had a substantially higher success rate compared with those who interviewed virtually. This is consistent with a previous survey of medical students, which found that the average number of programs applied to for all applicants (for all specialties) rose from 35.4 to 47.7 after the COVID-19 pandemic while interview invitations received remained steady at 16.8 and 16.3.^3^ That said, residents who interviewed virtually attended a higher percentage of interviews compared with those who interviewed in-person. Considering that most of the interviews involve a preinterview social the evening before a full day of interviews, this means that the in-person group likely spent roughly 3 days total per interview (1 day for arrival and social, 1 day for interviews, 1 day for travel after interviews) and attending 2 interviews on consecutive days was likely impossible. Conversely, those who interviewed virtually would more easily be able to attend back-to-back interviews.

Even before the COVID-19 pandemic, the number of residency applications submitted per applicant had been steadily rising, both across all specialties^4^ and within neurology specifically.^5^ It seems that the shift to virtual interviews during the pandemic only accelerated this trend. While it makes sense from an applicant perspective to apply broadly to maximize the chances of a successful match, this rise in applications adds a significant workload to programs, who must review more applications before inviting applicants for interviews. It also increases the chances of otherwise strong applicants not receiving interview invitations, whether because the program perceived other applicants as being more interested or simply because the strong applicants were overlooked as one of the vast number of applications received.

One of the biggest differences between the in-person and virtual interview groups was cost. The in-person group spent a median of $2,750 for interviews, mainly related to travel expenses. Meanwhile, the virtual group spent a median of $40, likely for upgraded camera systems for their computers. This cost difference has been shown numerous times before, including 1 survey of applicants for surgical specialties in the prepandemic era, where applicants spent a mean of over $5,000 on interview costs.^6^ A study of fourth-year medical students applying for residency in any specialty at the University of Kansas showed an 80% reduction in interview costs ($3,566 per applicant) during the 2020–2021 interview season compared with previous years.^7^ Another study showed that neurosurgery applicants spent an average of $786 in 2021 (during the fully virtual season) vs $4,511 in 2022, when many interviews shifted back to in-person.^8^ This study looks specifically at neurology neurolgoy applicants and shows concordance with existing literature regarding interview cost.

Owing to the high costs associated with in-person interviews, significantly more respondents in the in-person group needed to incur debt for these expenses. Previous studies have shown an even higher percentage of applicants needing to accrue debt for interviews, ranging from 33.8% of applicants for integrated plastic surgery^9^ to 52% of applicants for otolaryngology.^10^ One additional study that surveyed applicants across all specialties during the 2016 match cycle found that 71% of applicants accrued extra debt for interviews.^11^

Considering the astronomical costs of medical school attendance (average medical school loan balances are over $190,000^12^), the additional costs of in-person interviews place an extra burden on an already-strained economic picture. This has clear implications for equity among residency applicants because those with more financial resources at their disposal could conceivably attend more in-person interviews, thereby increasing their chances of a successful match, whereas those with less financial resources would be limited to only attend those interviews that they could afford. Indeed, 28% of the in-person applicants did not attend at least 1 interview they were offered because of cost while only 2.9% of the virtual applicants reported the same. International medical graduates would likely face even higher out-of-pocket costs for in-person interviews because they would likely need to travel longer distances than US medical graduates. Virtual interviews seem to be a way to ensure that there is no unfair advantage for applicants with more financial resources at their disposal. Considering that any extra financial burden would likely disproportionately affect applicants from historically marginalized groups, such as those under-represented in medicine, we must take care to ensure that future changes to the interview process take equity into account.

One of the major arguments for in-person interviews is that both programs and applicants can get a better sense of each other. Our survey results seem to bear this out, at least from the applicant perspective. Significantly more in-person applicants felt either very or extremely confident in their ability to get a feel for the culture of a program and its location, compared with the virtual applicant group. Because an in-person interview requires a visit to the program's location by definition, this difference is not surprising. It is possible that virtual interviews could lead to recruitment disadvantages for programs located outside well-known locations because the intangible benefits of living in a relatively unknown locale are not as easily conveyed through the virtual format.

These results echo similar results in other specialties. A recent study investigating the first year of virtual residency interviews for general surgery found that applicants had difficulty discerning program culture in the virtual format.^13^ Similarly, a survey of surgical program directors found that they had significant difficulty assessing an applicant's overall fit with their program by virtual interviews.^14^ Another study of program directors across various specialties echoed these results: nearly half of all respondents felt that they could not confidently assess an applicant's fit and over half felt that it was challenging to gauge an applicant's genuine interest or to represent their program well using a virtual platform.^15^ Considering that matching at a neurology residency program entails the applicant spending the next 4 years of their life at that program, the ability of both the applicant and the program to assess fit is of tantamount importance. An ill-informed residency match could have significant negative consequences down the road for both applicants and programs because it could lead to higher rates of resident burnout and program dissatisfaction and could negatively affect overall training and patient care.

Aside from the interview itself, one way in which applicants and programs generally ascertain fit is through a social gathering the evening before the interview. This most often takes the form of a dinner with current residents, which allows both applicants and residents to gauge one another in a social setting. For virtual interviews, these preinterview socials have presented a particular challenge—how to best show resident life outside work in a virtual setting? The difference in effectiveness between in-person vs virtual social gatherings was borne out on our survey, with significantly more in-person applicants feeling confident in the effectiveness of these preinterview gatherings compared with the virtual applicants. Indeed, most of the difference between in-person and virtual interviews in gauging the culture of a program was driven by the difference in these preinterview socials.

Although our survey did not directly address this, one point that must be made here is the murkiness of the word “fit” and the various meanings it has been given over the years. We must be careful that fit does not actually refer to unconscious biases for or against certain groups. A 2019 article discussed how some programs use fit to describe applicants sharing a similar background with current faculty and trainees while others use it to emphasize personality traits.^16^ We must ensure that an overemphasis on fit between the program and applicant does not lead to a reduction in diversity within programs and within the world of neurology as a whole. One potential way around such emphasis on subjectivity is to develop a trait-based system for evaluating applicants, which should help to control for unconscious biases on the part of the rater. Trait-based systems have higher inter-rater reliability in judging surgical residency applications than a system reliant on a “global rating of application elements.”^17^ We believe that programs should strongly consider a transparent and trait-based evaluation system for all applicants, as a way to ensure that fit and ultimate ranking are less susceptible to unconscious bias.

The concept of “fit” also introduces bias on the part of the applicant. Perhaps, virtual interviews can allow for a more standardized interview process, eliminating the influence of outside factors (jetlag, quality of food provided, interruptions in travel, etc) and allowing programs to be judged solely on their educational content. By allowing applicants to interview at more programs, virtual interviews may also provide a larger sample size and, therefore, provide applicants with more meaningful data from which to generate their rank lists.

The final question of our survey asked “if given the choice, would you prefer interviews be in-person or virtual?” Overall, respondents were likely to prefer the interview type they themselves experienced, although those who interviewed virtually seemed to feel less strongly about this. That said, 58.3% of the total respondents would prefer in-person interviews. Although this difference was not statistically significant, it did show a trend toward in-person interviews among all residents. It seems that most current neurology residents believe that the advantages of assessing program culture outweigh the higher costs of in-person interviews. Perhaps, this is seen as an investment of sorts because the residents are comfortable spending money to feel more certain of the place they will live and the program where they will train for the ensuing 4 years. The crux of the issue lies in the actual cost, however—surely, there must be some limit of money at which it is no longer “worth it” to interview in-person. The tradeoff between money spent and confidence in one's ranking decision likely has a sweet spot where both factors are optimized. Perhaps, medical schools could incorporate more financial literacy courses to allow applicants a better understanding of this tradeoff before the interview process.

As stated above, virtual interviews are substantially cheaper for the applicants but have drawbacks related to determining the culture or overall “fit” of a program. We will attempt to propose some innovative solutions to address each of these challenges within a given interview format.

Clearly, in-person interviews are significantly more financially burdensome. Increasing funding availability for all residency applicants would help alleviate this, although it may be far-fetched. While certain programs may have extra funds to offset the costs of interviews for their applicants, many other programs, health systems, and specialties likely do not have this funding. Likewise, it is unlikely that medical schools could provide such funding for their students. Governmental aid, in the form of loans, tax subsidies, or grants, could be explored but would likely further exacerbate inequities faced by international medical graduates (who would be presumably less likely to qualify for such aid). Short of providing universal financial aid, another potential solution to the financial burden of in-person interviews would be to allow applicants the choice of whether to interview virtually or in-person. The fear of such a combined method is that programs would favor applicants who interviewed in-person, so this solution would require a transparent process to mitigate bias. One option would be to have a third party, such as the program's Designated Institutional Official, review the ultimate rank order lists to ensure that there is no apparent bias toward the in-person applicants. Another option may be development of a transparent “scoring rubric” that is able to eliminate the impact of in-person interviewing on an applicant's final rank order placement. Regardless, full transparency would be an essential component of this type of solution.

The major drawback of the virtual interview process is the perceived inability of applicants to assess the culture of a program or their own “fit” for the program. We could potentially alleviate this by further encouraging nonevaluative second look opportunities for applicants to visit a specific program after interviews have been completed (and after the program has submitted and certified their own rank order list). In this case, there could be a staggered deadline for submission of rank order lists, with programs being required to submit theirs some number of weeks before applicants. This would alleviate any concerns about bias toward those applicants who travel to the program but has the drawback of preventing the program from being able to evaluate applicants based on in-person interactions. We could also work to improve the virtual interview experience to better demonstrate the program's culture. Future studies could examine what exactly is missing in the virtual interview landscape and how programs could best add this missing piece. As an example, perhaps, certain ways of conducting the preinterview social with current residents are more effective than others and those effective strategies could be shared with programs nationwide. It is possible that burgeoning technologies such as virtual reality could even be used to better approximate the in-person experience, although it is conceivable that this might reintroduce a significant financial burden. Ultimately, if virtual interviews continue into the future, both programs and applicants are likely to continue to develop innovative ways to improve the experience on both ends, and it is our hope that these solutions would be shared with all.

Our survey was completed by 60 neurology residents across 4 years of training at 5 residency programs throughout the United States. Although we did have many statistically significant results, a larger number would likely have yielded even more information, especially regarding the ultimate question of which interview mode the residents would prefer. There is also some potential recall bias because the respondents who interviewed in-person by default would have undergone the process years before those who interviewed virtually. We likewise did not take into account the demographic data of our cohort, and we did not track the level of financial resources our residents had available during the residency interview process.

In addition, 4 of the 5 programs surveyed are in the eastern time zone, where distance between cities and programs is shorter and, therefore, travel costs are potentially lower. If our survey included more programs in the central, mountain, and western time zones, where there is more distance and presumably a higher travel cost between cities, it is possible that the cost difference between in-person and virtual interviews would have been even greater. We did not track the geographic origin of the residents nor did we track their medical school status (e.g., US allopathic graduates vs US osteopathic graduates vs international medical graduates). It is possible that a larger cohort with subgroup analyses would show significant differences in opinions among applicants from these varied geographic, cultural, and economic backgrounds. Furthermore, our survey was not externally validated because we created it specifically for this project. The survey questions themselves potentially introduced framing bias in how certain questions were asked and in excluding “neutral” or “no opinion” as possible answers. We did not have demographic questions out of privacy concerns, but future studies with a larger number should certainly include these as important potential confounders and descriptive variables. Future studies could also include more open-ended questions with qualitative analysis and could specifically ask whether applicants were ultimately satisfied with their eventual match.

The logical next step in this line of study would be a similar survey aimed at current neurology residency program directors, seeking their opinions on the same question of in-person vs virtual interviews. A previous study discussed above did ask this question of program directors across all specialties^15^ but did not focus on neurology specifically. Another factor for future study is the return on investment of in-person interviews: in other words, just how much money is the in-person experience worth? While this would be a difficult answer to quantify and would likely vary from applicant to applicant, it would be helpful in informing future interview choices.

As the COVID-19 pandemic subsides, many programs and specialty organizations are left with a conundrum: do we return to in-person residency interviews or do we stick with the virtual interview format? Our survey shows that in-person interviews were associated with a decreased application burden, greater interview success rate, and a better feel for the overall fit of a program but were also associated with thousands of extra dollars spent per applicant. The ultimate question is as follows: are the advantages in determining fit and managing application bloat worth the significant costs of in-person interviews? It seems that for many residents, the answer is yes. Whatever the choice, there should be a standard for any given specialty (i.e., all neurology programs should offer either virtual or in-person interviews). Our data would also strongly suggest that if interviews return to an in-person format, programs should try to alleviate any economic inequities among applicants, by way of paying for accommodations and meals and potentially even providing travel vouchers. Programs and specialties that do choose to return to in-person interviews should provide explicit rationale for their choice to do so.

Ultimately, with the social isolation of the COVID-19 pandemic largely mitigated, the return of in-person interviews seems inevitable. We strongly recommend that whatever approach is taken moving forward, it be performed in a thoughtful and evidence-based manner, with an aim to eliminate bias, promote equity, and provide full transparency wherever possible.

## Appendix Authors

**Table:**

Name	Location	Contribution
William Alexander Dalrymple, MD	Department of Neurology, University of Virginia, Charlottesville	Drafting/revision of the manuscript for content, including medical writing for content; major role in the acquisition of data; study concept or design; analysis or interpretation of data
Robin Ulep, MD	Department of Neurology, Icahn School of Medicine at Mount Sinai, New York, NY	Drafting/revision of the manuscript for content, including medical writing for content; major role in the acquisition of data; study concept or design; analysis or interpretation of data
Jeffrey B. Ratliff, MD	Department of Neurology, Thomas Jefferson University, Philadelphia, PA	Drafting/revision of the manuscript for content, including medical writing for content; major role in the acquisition of data; study concept or design; analysis or interpretation of data
Joseph Carrera, MD	Department of Neurology, University of Michigan Medical School, Ann Arbor	Drafting/revision of the manuscript for content, including medical writing for content; major role in the acquisition of data; study concept or design; analysis or interpretation of data
Alan Wang, MD	Department of Neurology, University of Arizona College of Medicine – Phoenix	Drafting/revision of the manuscript for content, including medical writing for content; major role in the acquisition of data; study concept or design; analysis or interpretation of data
James T. Patrie, MS	Department of Public Health Sciences, University of Virginia, Charlottesville	Drafting/revision of the manuscript for content, including medical writing for content; analysis or interpretation of data
Andrew M. Southerland, MD	Department of Neurology, and Department of Public Health Sciences, University of Virginia, Charlottesville	Drafting/revision of the manuscript for content, including medical writing for content; major role in the acquisition of data; study concept or design; analysis or interpretation of data

## Glossary

COVID-19: coronavirus disease 2019
IRB: institutional review board
UVA: University of Virginia

## References

[1] Ponterio JM, Levy L, Lakhi NA. Evaluation of the virtual interviews for resident recruitment due to COVID-19 travel restrictions: a nationwide survey of US senior medical students. Fam Med. 2022;54(10):776-783. doi:10.22454/FamMed.2022.592364 36350742

[2] Schuyler E, Matthews R, Kung D, et al. American Academy of Neurology Consensus Statement on the 2023-2024 Residency/Fellowship Application Cycle. AAN; 2023. Accessed March 7, 2024. aan.com/siteassets/home-page/tools-and-resources/academic-neurologist--researchers/program-coordinator-resources/23-neuro-residency-interview-statement.pdf.

[3] Meyer AM, Hart AA, Keith JN. COVID-19 increased residency applications and how virtual interviews impacted applicants. Cureus. 2022;14(6):e26096. doi:10.7759/cureus.26096 35875277 PMC9298600

[4] Weissbart SJ, Kim SJ, Feinn RS, Stock JA. Relationship between the number of residency applications and the yearly match rate: time to start thinking about an application limit? J Grad Med Educ. 2015;7(1):81-85. doi:10.4300/JGME-D-14-00270.1 26217428 PMC4507934

[5] ERAS Statistics: 2019-2023 ERAS Seasons, Neurology. AAMC; 2023. Accessed November 29, 2023. aamc.org/media/39846/download.

[6] Gordon AM, Pulford C. Assessing the finances of applying to surgical residency in 2019-2020: a US nationwide surgical specialties comparison. J Grad Med Educ. 2023;15(5):558-563. doi:10.4300/JGME-D-23-00274.1 37781430 PMC10539139

[7] Nilsen K, Walling A, Johnson M, et al. The impact of virtual interviewing during the COVID-19 pandemic on the residency application process: one institution's experience. Acad Med. 2022;97(10):1546-1553. doi:10.1097/ACM.0000000000004761 36198163

[8] Mehkri Y, Pierzchajlo N, Kemeness C, et al. A cost analysis of medical students applying to neurological surgery residency: an analysis of the Texas STAR database. J Clin Neurosci. 2023;117:151-155. doi:10.1016/j.jocn.2023.09.031 37816269

[9] Sarac BA, Rangwani SM, Schoenbrunner AR, Drolet BC, Janis JE. The cost of applying to integrated plastic surgery residency. Plast Reconstr Surg Glob Open. 2021;9(1):e3317. doi:10.1097/GOX.0000000000003317 33564569 PMC7859384

[10] Polacco MA, Lally J, Walls A, Harrold LR, Malekzadeh S, Chen EY. Digging into debt: the financial burden associated with the otolaryngology match. Otolaryngol Head Neck Surg. 2017;156(6):1091-1096. doi:10.1177/0194599816686538 28116996

[11] Fogel HA, Liskutin TE, Wu K, Nystrom L, Martin B, Schiff A. The economic burden of residency interviews on applicants. Iowa Orthop J. 2018;38:9-15. Accessed November 30, 2023. ncbi.nlm.nih.gov/pmc/articles/PMC6047386/. 30104919 PMC6047386

[12] McKillip R, Ernst M, Ahn J, Tekian A, Shappell E. Toward a resident personal finance curriculum: quantifying resident financial circumstances, needs, and interests. Cureus. 2018;10(4):e2540. doi:10.7759/cureus.2540 29951347 PMC6019332

[13] Finney N, Stopenski S, Smith BR. Applicant perspectives of virtual general surgery residency interviews. Am Surg. 2022;88(10):2556-2560. doi:10.1177/00031348221103658 35610972

[14] Asaad M, Elmorsi R, Ferry AM, Rajesh A, Maricevich RS. The experience of virtual interviews in resident selection: a survey of program directors in surgery. J Surg Res. 2022;270:208-213. doi:10.1016/j.jss.2021.09.011 34706297

[15] Ponterio JM, Levy L, Lakhi NA. Evaluation of the virtual interview format for resident recruitment as a result of COVID-19 restrictions: residency program directors' perspectives. Acad Med. 2022;97(9):1360-1367. doi:10.1097/ACM.0000000000004730 35507455

[16] Shappell E, Schnapp B. The F word: how “fit” threatens the validity of resident recruitment. J Grad Med Educ. 2019;11(6):635-636. doi:10.4300/JGME-D-19-00400.1 31871561 PMC6919185

[17] Gawad N, Younan J, Towaij C, Raiche I. Comparing 2 approaches for the file review of residency applications. J Grad Med Educ. 2021;13(2):240-245. doi:10.4300/JGME-D-20-00619.1 33897958 PMC8054590

